# A new species of *Rhaptopetalum* (Lecythidaceae) from south-western Gabon

**DOI:** 10.3897/phytokeys.128.34640

**Published:** 2019-07-23

**Authors:** David Kenfack, Diosdado Ekomo Nguema

**Affiliations:** 1 Forest Global Earth Observatory (ForestGEO), Smithsonian Tropical Research Institute, Apartado Postal 0843-03092, Panamá, República de Panamá Smithsonian Tropical Research Institute Panamá Panama; 2 Physical address: Department of Botany, National Museum of Natural History - MRC 166, P.O. Box 37012, Washington, DC 20013-7012, USA National Museum of Natural History Washington United States of America; 3 Gabon Biodiversity Program, Centre for Conservation and Sustainability, Smithsonian Conservation Biology Institute, BP 48, Gamba, Gabon Smithsonian Conservation Biology Institute Gamba Gabon

**Keywords:** ForestGEO, Gabon, IUCN Red List, new species, permanent plot, Rabi, rainforest, taxonomy

## Abstract

*Rhaptopetalumrabiense* Kenfack & Nguema, **sp. nov.** from the Rabi forest in south-western Gabon is described, illustrated and assigned a provisional conservation status of “Critically Endangered”. An identification key to the five Gabonese species of *Rhaptopetalum* is also provided.

## Introduction

*Rhaptopetalum* Oliv. is a genus of 12 species of trees, mostly confined in the Gulf of Guinea, with only one species occurring in west Africa (Cheek et al. 2002; Prance and Jongkind 2015). The genus was first described in 1865 by the English botanist Daniel Oliver (1865), as a member of the family Scytopetalaceae. Recent molecular phylogenetic analyses (Morton et al. 1997; Mori et al. 2007) showed that Scytopetalaceaeform a monophyletic group with Lecythidaceae. Yet, opinions still diverge about either keeping the Scytopetalaceae as a separate family (Takhtajan 2009; Reveal 2011) or as a subfamily within Lecythidaceae (Mabberley 2008; APG 2016). Here, we consider *Rhaptopetalum* as a member of the Lecythidaceae s.l. Eight species of *Rhaptopetalum* are cited in the *Flore du Gabon* account of Scytopetalaceae (Letouzey and White 1978). However, because no specimen citation was provided for four of them (*R.breteleri* R. Letouzey, *R.depressum* R. Letouzey, *R.roseum* (Gürke) Engler and *R.sessilifolium* Engler), their presence in Gabon remains doubtful. Hence, only four species are currently recognised in Gabon (Prance and Jongkind 2015).

In 2010, we established a 25-ha permanent plot in the rainforest of south-western Gabon to study the long-term dynamics of this forest (Memiaghe et al. 2016). The methods included the challenging task of identifying to species level hundreds of thousands of sterile trees and saplings with diameter at breast height (dbh) ≥ 1cm. Amongst the 175,830 trees recorded in the plot, 299 were assigned to *Rhaptopetalum* Oliv., based on the short petiolate leaves with punctate lamina, the flowers with articulated pedicel and cupuliform calyx, the stamens attached to the base of the pseudocorolla, the poricidal anthers, the short filaments and the pubescent seeds. The identification of the Rabi material, using the key in the recent revision of the African Lecythidaceae (Prance and Jongkind 2015), was problematic from the second couplet. The species does not fit either of the leads 3 or 4, because the ovary has few (generally 1) ovules per locule, is dome-shaped, while the fruit surface is not angled. Hence, following the lead 4, the Rabi species is close to the Gabonese *Rhaptopetalumbelingense* Letouzey with its dome-shaped ovary, its entire calyx margin, its apex placentation and smooth fruits. Following lead 3, it also resembles the Democratic Republic of the Congo *R.evrardii* R. Germain with its puberulous midrib, red petals and cupuliform calyx and the pedicel not articulated immediately below the calyx. However, the Rabi material also presents a suite of unique characters outlined below (Table 1) and that allows us to describe it as new to science.

**Table 1.** Comparison of discriminant characters amongst *Rhaptopetalumrabiense*, *R.evrardii* and *R.belingense*.

	***R.rabiense***	***R.evrardii***	***R.belingense***
Indumentum of young branches	Puberulous	Puberulous	Glabrous
Shape of young branches	Not angular	Angular	Not angular
Lamina length (cm)	7–20	18–28	15–18
Lamina width (cm)	3–9	8–12	8–10
Pedicel length (mm)	5–7	4–5	3
Pedicel articulation	1 mm below the calyx	about 1 mm below the calyx	Directly below the calyx
Calyx margin	Entire	6–10 lobed	Entire
Calyx shape	Cupuliform	Cupuliform	Flattened patelliform
Pseudocorolla length (mm)	3–5	7–8	8
Number of ovules per locule	1	2 or 3	Many
Fruit surface	Smooth	Ridged	Smooth
Fruit diameter (mm)	15–20	10–12	15

## Taxonomic treatment

### Key to the Gabonese species of *Rhaptopetalum*

**Table:** 

1	Leaf lamina exceeding 18 cm long and 10 cm wide	**2**
–	Leaf lamina up to 18 cm long and 10 cm wide	**3**
2	Ovary conical, pedicel 3—5 mm long, placentation axile	***R.sindarense***
–	Ovary dome-shaped, pedicel 8–10 mm long, placentation apical	***R.pachyphyllum***
3	Leaf base cuneate, slightly decurrent onto petiole	***R.coriaceum***
–	Leaf base rounded	**4**
4	Young branches glabrous, petiole 5—7 mm long, ovary loci multi-ovulate	***R.belingense***
–	Young branches puberulous, petiole 2—4 mm long, ovary loci uni-ovulate	***R.rabiense***

#### Rhaptopetalum rabiense

Taxon classificationPlantaeEricalesLecythidaceae

Kenfack & Nguema sp. nov.

a035ecfa-4262-5f5e-8897-7ef9784033ae

urn:lsid:ipni.org:names:77200426-1

Figs 1 , 2

##### Type.

GABON. Ogooué Maritime, 25-ha Rabi Forest plot, 1°55'37.57"S, 9°52'50.66"E, 23 m alt., 27 Aug 2014 (fl), *Nguema et al. 2825a* (holotype: LBV, isotypes: BR, MO, US, K, P)

##### Diagnosis.

*Rhaptopetalumrabiense* is similar to *R.belingense* by its dome-shaped ovary, its entire calyx margin, its apex placentation and smooth fruits, but differs by its pubescent (vs. glabrous) young branches, its longer pedicel (5–7 mm vs. 3 mm) articulated 1 mm below the calyx (vs. articulated directly below the calyx), and its uni-ovulated loci (vs. multi-ovulated) (Table 1).

##### Description.

*Tree* 4–6 m tall, bole cylindrical, to 20 cm diameter at 1.3 m aboveground; bark pale brown, slash fibrous, pink in the outer part, yellowish in the inner part; young branches slender, rusty brown, densely puberulous, conspicuously lenticellate. *Leaves* distichous; petiole 2–4(6) × 2 mm, densely puberulous; lamina obovate to elliptic, coriaceous, abundantly punctate, 8–18(21) × (2.5)5–9 cm, acuminate to broadly acute at apex, slightly unequal and rounded at base; margin entire; midrib conspicuous and flattened above, prominent and minutely puberulous beneath; secondary veins 8–11 pairs, plane above, prominent beneath, arching and joined towards the margin of the lamina. *Inflorescence* ramiflorous, axillary and supra-axillary, fasciculate, with 1–8 flowers. *Bracts* ovate, about 1 mm long. *Flower bud* ovoid to globose-oblong, rounded at apex, (2.5)3–4 mm long, pink to red directly above the calyx rim, light pink to whitish towards the apex. Pedicel in flower buds (2.5)3–5 mm, but generally 5–7 mm long in opened flowers, yellow-green, minutely puberulous to glabrescent, articulate directly beneath calyx. Calyx cupuliform, yellow-green, glabrescent to puberulous, the margin entire, 2–2.5 mm in diameter on the rim, receptacle about 1 mm long. Pseudocorolla fleshy, splitting into (3)4 lobes 2.5–4 mm long oblong to ovate lobes. *Stamens* 35(37), the filament light pink to whitish, about 0.3 mm, the poricidal anthers bright yellow, slightly arched towards the centre of the flower, 2–2.5 mm long; ovary superior, globose, about 1 mm high and 1.5 mm diameter, 3–4-locular, each locule with 1 or 2 axile pendulous ovules. Style 3–4.5 mm long. *Fruit* a globose capsule, green, smooth, 15–20 mm diam., fruiting pedicel 5–7 mm long, seeds 8–12 × 5–8 mm.

**Figure 1.**
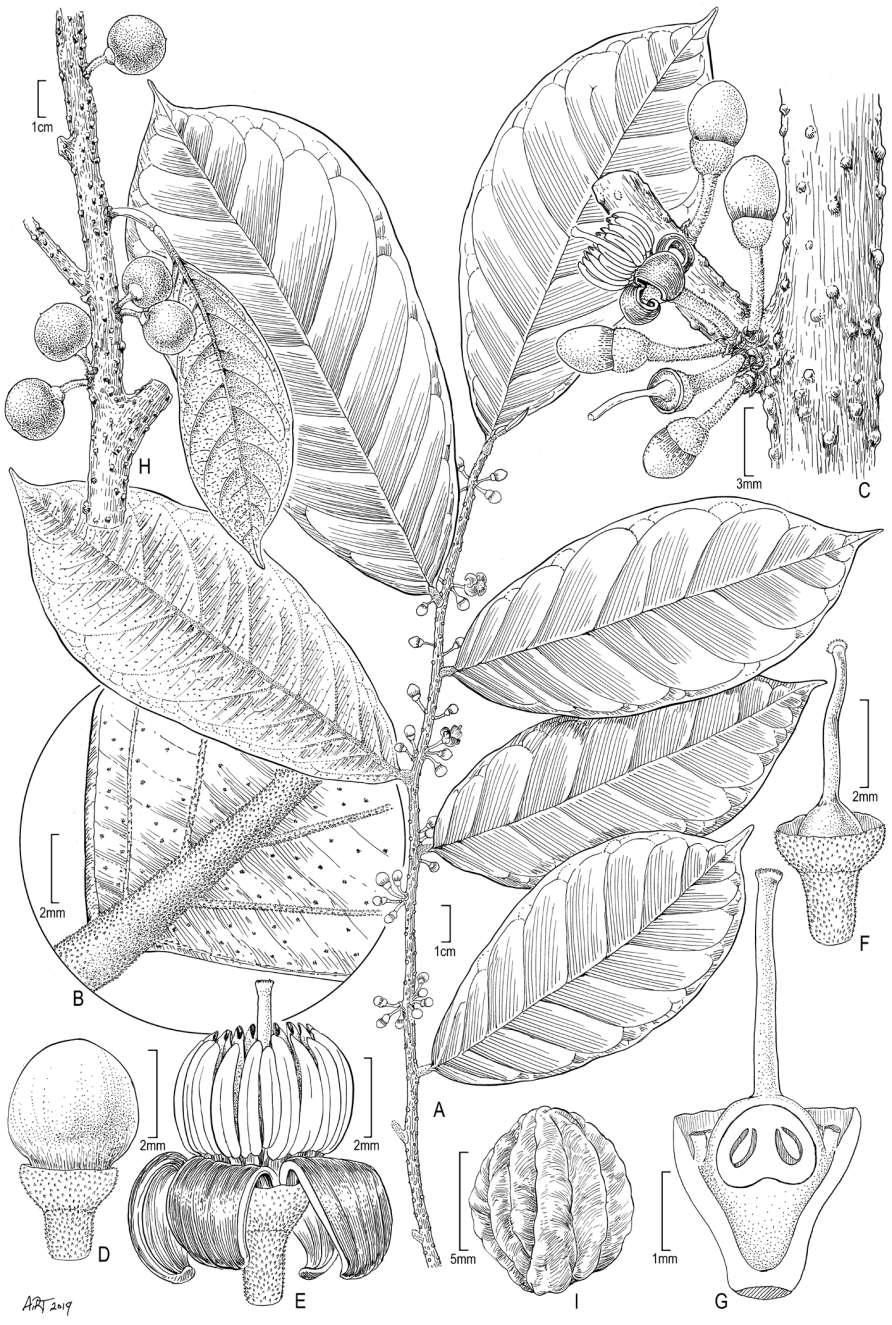
*Rhaptopetalumrabiense***A** flowering twig **B** close-up of the lower surface showing the indumentum and the punctate lamina **C** detail of inflorescence and stem showing lenticels **D** flower bud **E** opened flower **F** flower with pseudocorolla and stamens removed showing superior ovary **G** longitudinal section of **F** showing pendulous ovules **H** fruiting branch.

**Figure 2.**
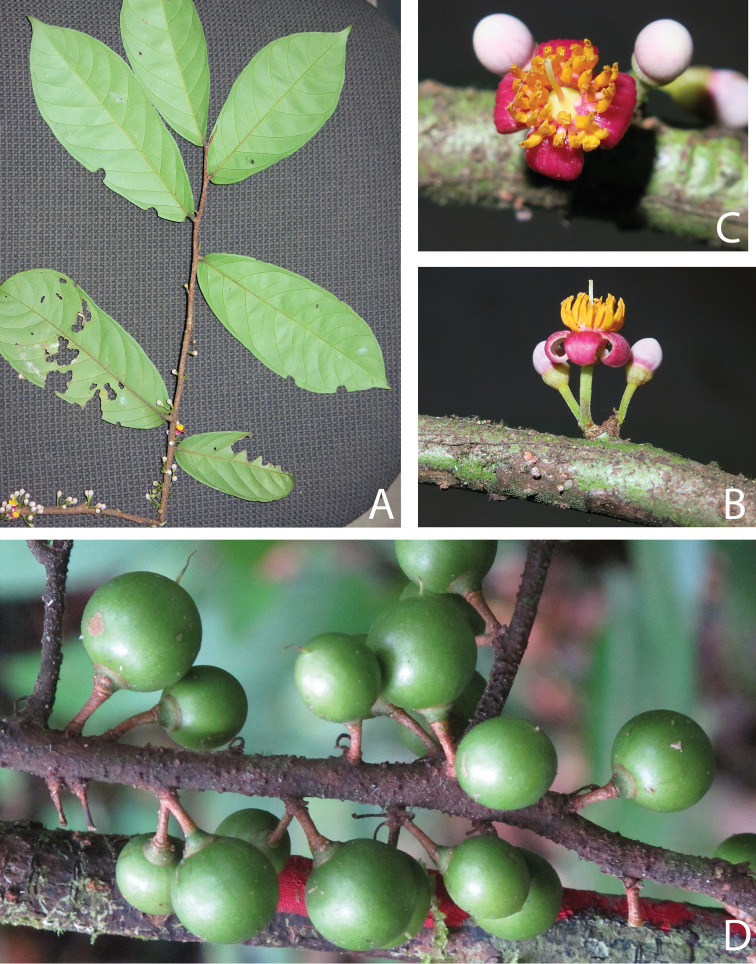
*Rhaptopetalumrabiense***A** flowering branch **B** lateral view of the flower **C** flower view from above showing the poricidal anthers and the gynoecium **D** fruiting branch. Photographs by Diosdado Nguema.

##### Phenology.

Flowering August–October, Fruiting September–December.

##### Geographic distribution.

**Figure 3.**
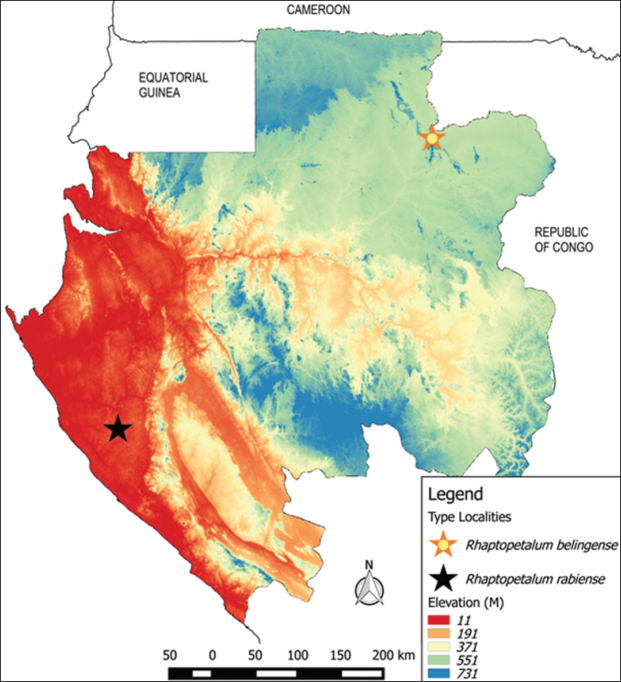
Type locations of *Rhaptopetalumrabiense* and the closely related species *R.belingense* in Gabon.

*Rhaptopetalumrabiense* is only known from the type locality, the Rabi forest (Figure 3).

##### Habitat.

*Rhaptopetalumrabiense* grows in old growth forest, on both terra firme dry and wet depressions, with elevation 20–50 m.

##### Additional specimens examined.

GABON. Ogooué Maritime: Rabi, 25-ha permanent plot, 24 m elev., 1°55'28.1"S, 9°52'48.26"E, 21 August 2013 (st), *Nguema et al. 1743*; Rabi, 25-ha permanent plot, 50 m elev., 1°54'51.36"S, 9°52'41.56"E, 28 October 2013 (st), *Nguema et al*. 1922; Rabi, 25-ha permanent plot, 50 m elev., 1°55'27.09"S, 9°52'41.56"E, 5 November 2013 (st), *Nguema et al. 2057*; Rabi, 25-ha permanent plot, 32 m elev., 1°55'37.57"S, 9°52'50.66"E, 2 September 2014 (fl), *Nguema et al. 2825B*; Rabi, 25-ha permanent plot, 32 m elev., 1°55'37.57"S, 9°52'50.66"E, 2 September 2014 (fr), *Nguema et al. 2825C*; Rabi, 25-ha permanent plot, 32 m elev., 1°55'30.22"S, 9°52'42.18"E, 23 September 2014 (fl), *Nguema et al. 2832*; Rabi, 25-ha permanent plot, 32 m elev., 1°55'30.35"S, 9°52'41.79"E, 2 October 2014 (fl), *Nguema et al. 2833*; Rabi, 25-ha permanent plot, 61 m elev., 1°55'36.44"S, 9°52'46.61"E, 2 December 2014 (fr), *Nguema et al. 2926*.

##### Preliminary conservation status.

The conservation status of *Rhaptopetalumrabiense* was evaluated using the IUCN Red List Categories and Criteria Version3.1 (IUCN 2012). The extent of occurrence (EOO) and the area of occupancy (AOO), estimated using the web Geospatial Conservation Assessment Tool or GeoCAT (Bachman et al. 2011) and the auto-value cell size length of 2 km, were 0.214 km^2^ and 8.00 km^2^, respectively. These two values meet the criteria B1 (EOO < 100 km^2^) and B2 (AOO < 10 km^2^) for Critically Endangered, following the IUCN Red List Categories and Criteria Version 3.1 (IUCN 2012). The species is not under legal protection. The type locality is an oil and gas production field and, at the same time, a logging concession. These activities are likely to result in its population reduction and/or in a fragmentation of its range. *R.rabiense* in the studied plot has 299 individuals with dbh ≥ 1cm (12 individuals per hectare) and seems to be regenerating, based on diameter size class distribution. It is known only from its type locality and has not been recorded in the adjacent national parks of Loango National Park on the west and Moukalaba Doudou National Park on the east. We therefore assess *R.rabiense* with the preliminary IUCN Red List status of Critically Endangered CR B12ab(iii).

## Supplementary Material

XML Treatment for Rhaptopetalum rabiense
